# Germline heterozygous *DDX41* variants in a subset of familial myelodysplasia and acute myeloid leukemia

**DOI:** 10.1038/leu.2016.124

**Published:** 2016-05-20

**Authors:** S R Cardoso, G Ryan, A J Walne, A Ellison, R Lowe, H Tummala, A Rio-Machin, L Collopy, A Al Seraihi, Y Wallis, P Page, S Akiki, J Fitzgibbon, T Vulliamy, I Dokal

**Affiliations:** 1 Centre for Genomics and Child Health, Barts and The London School of Medicine and Dentistry, Queen Mary University of London, Barts NHS Trust, London, UK; 2 Birmingham Women's NHS Foundation Trust, Birmingham, UK; 3 Centre for Haemato-Oncology, Barts Cancer Institute, Queen Mary University of London, London, UK

Myelodysplasia (MDS) and acute myeloid leukemia (AML) are mostly sporadic hematopoietic stem cell clonal disorders. However, there are rare occurrences of familial MDS/AML where there are two or more affected cases in the same family. To date, germline heterozygous mutations have been identified in 10 genes (*RUNX1*, *CEBPA*, *TERC*, *TERT*, *GATA2*, *SRP72*, *ANKRD26*, *ACD*, *ETV6* and *DDX41*)^1, 2, 3, 4, 5, 6, 7, 8, 9, 10^ associated with familial MDS/AML. Over the last 15 years we have accrued 78 families in which there are at least two cases of bone marrow failure and at least one of whom has MDS or AML. We have undertaken a combination of whole-exome and targeted sequencing to characterize these families. The targeted sequencing uses a newly designed familial MDS/AML gene panel that includes the above 10 listed genes. This analysis has enabled us to identify four families harboring heterozygous germline *DDX41* (DEAD-box helicase 41) variants (Figures 1a–d); three families have novel frameshift variants (c.155dupA, c.1586_1587delCA and c.719delTinsCG) and the fourth family has a recurrent missense variant in the initiation codon (c.3G>A, rs141601766) described previously by Lewinsohn *et al.*^11^ Collectively, these four families comprise seven cases of MDS and two cases of AML (age range, 40–70 years). These patients did not have any extra-hematopoietic features and therefore represent ‘pure' MDS/AML (Table 1).

At present, little is known about DDX41 function and its role in hematopoiesis. However, Polprasert *et al.*^10^ showed that the protein encoded by *DDX41* interacts directly with spliceosomal proteins and inactivation of tumor suppressors can occur once this interaction is disrupted. It is known that members of the DEAD/H box RNA helicase family can act as oncogenes or tumor suppressors in other cancers, depending on the specific protein interactions.^12^ In addition, alterations in *DDX41* can cause exon skipping or exon retention in the RNA-splicing process resulting in alteration of specific genetic isoforms.^10^

Kirwan *et al.*^3^ demonstrated that familial MDS/AML patients with germline variants in *TERT* and *TERC* have significantly shorter telomeres compared with controls. To determine whether our group of ‘pure' MDS/AML patients with germline *DDX41* variants have a similar impact on telomere length, we measured peripheral blood telomere length by monochrome multiplex quantitative PCR method^13^ in our patients. Slightly shorter telomere length was found in this group of patients harboring germline *DDX41* variants compared with age-matched controls (*P*<0.05, Supplementary Figure S1). It will be important to investigate telomere length in additional patients with *DDX41* variants to substantiate these observations.

In Family 1 (Figure 1a), a novel heterozygous germline variant c.155dupA (p.Arg53Alafs*16 showed in Figure 1e) in *DDX41* was identified in the 49-year-old female index case (III-1) diagnosed with MDS, refractory anemia with excess blasts (RAEB). Sanger sequencing revealed that her maternal uncle and aunt who both developed RAEB also harbor this frameshift variant (individuals II-2 and II-3, respectively). There are two asymptomatic carriers (individuals II-1 and II-4), supporting previous observations that haploinsufficiency for *DDX41* shows variable penetrance.^11^ Further family history included her father (II-5) who died of chronic myeloid leukemia, unlikely to be related to the *DDX41* variant.

In Family 2 (Figure 1b), the index case is a 60-year-old male (II-1) with AML harboring a novel heterozygous frameshift variant c.719delTinsCG (p.Ile240Thrfs*108), predicted to cause truncation of the protein and consequent loss of function. His mother died of AML (I-1). Segregation analysis was not possible as there were no family samples available, however the variant allele frequency in the index case is 0.494 indicating a heterozygosity. This variant is located in the DEAD-box domain of DDX41, in a highly conserved motif that includes the ATP-binding site of DDX41 (Figure 1e).

The 58-year-old female index case in Family 3 (II-2 in Figure 1c) with MDS, has a novel frameshift deletion variant c.1586-1587delCA (p.Thr529Argfs*12) in the helicase domain of DDX41 (Figure 1e), which is again predicted to cause truncation of the protein. Her brother has tongue cancer (II-4), her mother has MDS (I-1) and her father has stomach cancer (I-2). In the absence of samples of the index case's parents, Sanger sequencing was undertaken on samples from her siblings and children. The siblings (II-3 and II-4) of the index case do not harbor the variant c.1586-1587delCA, whilst her daughter (III-2) is an asymptomatic carrier. This suggests that the index case and her mother (both with MDS) have disease associated with the *DDX41* variant, while the non-hematological cancers seen in her brother (II-4) and father (I-2) are unrelated to *DDX41*.

The index case of Family 4 (Figure 1d) is a 41-year-old female (II-1) diagnosed with MDS/RAEB. Her father (I-2) was also diagnosed with MDS at the age of 64 years. The heterozygous missense variant c.3G>A (p.Met1Ile—rs141601766, showed in Figure 1e) in *DDX41*, which segregated with disease in these two individuals, has been reported in The Exome Aggregation Consortium (ExAC) database in 6/117 464 alleles (http://exac.broadinstitute.org/, accessed 31 March 2016). Interestingly, both cases with the c.3G>A variant also carried a linked 5′-untranslated region variant (c.-44G>A showed in Figure 1d) previously observed by Lewinsohn *et al.*^11^ They also demonstrated that human embryonic kidney 293 cells (HEK-293) cells ectopically expressing the Met1Ile mutant protein used an alternative translation initiation site yielding a smaller DDX41 protein when compared with the full-length of 70 kDa. Their experiments suggest that this isoform may occur naturally and has an altered location.

The recurrence of the Met1Ile variant in the ExAC database poses an interesting question as to the causative role of *DDX41* variants in MDS/AML. Excluding any non-canonical and dubious calls in this database, loss of function (LOF) variants (including Met1Ile) are seen to occur at a cumulative frequency of 1 in 1189 people (46 LOF variants in an average of 109 354 alleles). This is in stark contrast to the few LOF variants reported in *RUNX1* (6), *CEPBA* (0), *GATA2* (0) and *ETV6* (1). We also note that in a screen of 1034 patients with MDS and secondary AML, 7 patients (1 in 148) had germline LOF variants in *DDX41* (ref. 10). These data indicate that rather than establishing a causal Mendelian link between germline LOF *DDX41* variants and MDS/AML, it is better to think of them as genetic risk factors. Comparing the frequency of LOF *DDX41* variants seen in MDS and secondary AML with the frequency seen in ExAC we obtain an odds ratio of 8.05 (*P*=5.65 × 10^−5^, Fisher's exact test). Allowing for a 1/100 probability of getting the disease, this would translate to a relative risk of 7.51. It is inevitable therefore, that MDS/AML driven by *DDX41* LOF variants will sometimes appear as familial.

In summary, we report on novel germline heterozygous *DDX41* variants exhibiting variable penetrance in families with MDS/AML and tendency to short telomeres. Our analysis suggests that rather than establishing a causal Mendelian link between *DDX41* germline LOF variants and MDS/AML it is appropriate to consider these as genetic risk factors.

## Figures and Tables

**Figure 1:**
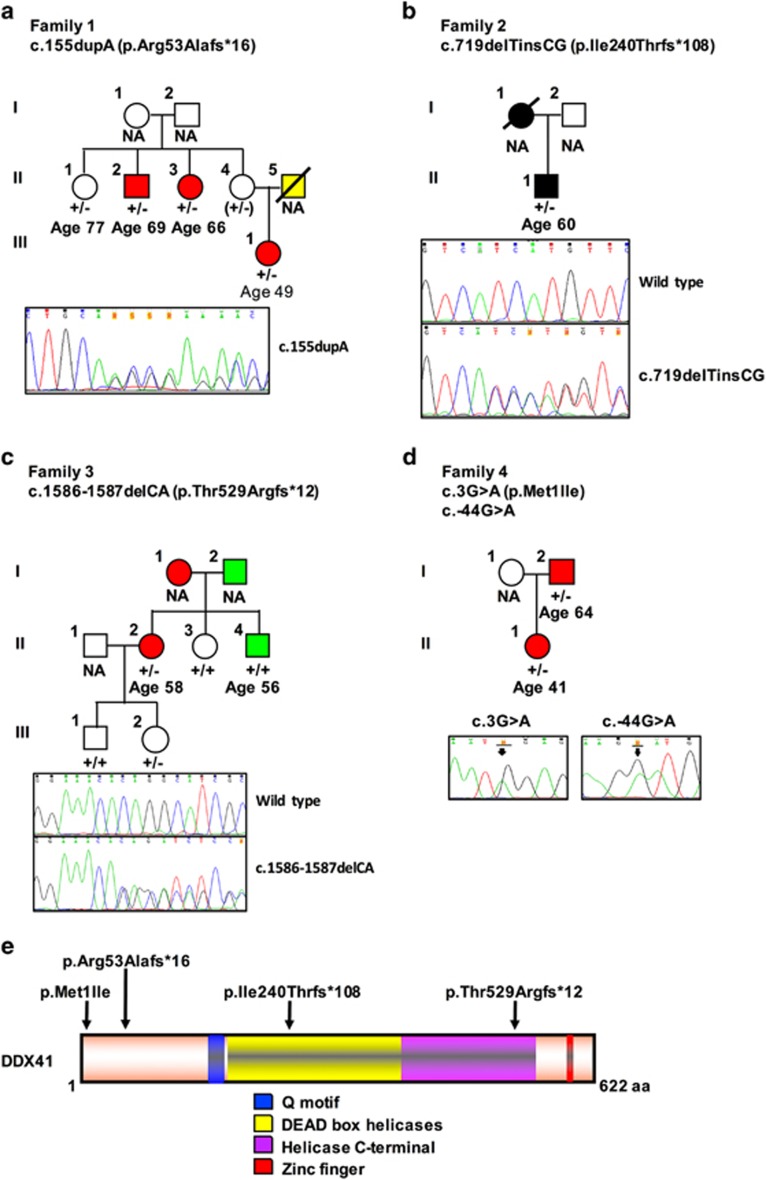
(**a**–**d**) Families with MDS–AML with variants in *DDX41*, their age at diagnosis and their respective Sanger sequencing traces. Affected individuals are colored as follows: red, MDS; yellow, CML; black, AML; and green, other non-hematological cancer. (**e**) Schematic of DDX41 protein showing the heterozygous variants identified in this study. CML, chronic myeloid leukemia.

**Table 1:** Characteristics and family history of index cases

*Family*	*Case*	*Age (years)*	*Diagnosis*	*Relationship to index*	*Nucleotide*	*Amino acid*
1	I-1	NA	Asymptomatic	Grandmother	NA	NA
	I-2	NA	Asymptomatic	Grandfather	NA	NA
	II-1	77	Asymptomatic	Maternal aunt	c.155dupA	p.Arg53Alafs*16
	II-2	69	MDS	Maternal uncle	c.155dupA	p.Arg53Alafs*16
	II-3	66	MDS	Maternal aunt	c.155dupA	p.Arg53Alafs*16
	II-4	NA	Asymptomatic	Mother	NA	NA
	II-5	NA	CML	Father	NA	NA
	III-1	49	MDS	Index case	c.155dupA	p.Arg53Alafs*16
2	I-1	NA	AML	Mother	NA	NA
	I-2	NA	Asymptomatic	Father	NA	NA
	II-1	60	AML	Index case	c.719delTinsCG	p.Ile240Thrfs*108
3	I-1	NA	MDS	Mother	NA	NA
	I-2	NA	Stomach cancer	Father	NA	NA
	II-1	NA	Asymptomatic	Husband	NA	NA
	II-2	58	MDS	Index case	c.1586-1587delCA	p.Thr529Argfs*12
	II-3	NA	Asymptomatic	Sister	NV	NV
	II-4	56	Tongue cancer	Brother	NV	NV
	III-1	NA	Asymptomatic	Son	NV	NV
	III-2	NA	Asymptomatic	Daughter	c.1586-1587delCA	p.Thr529Argfs*12
4	I-1	NA	Asymptomatic	Mother	NA	NA
	I-2	64	MDS	Father	c.3G>A	p.Met1Ile
					c.-44G>A Met1Ile	NA
	II-1	41	MDS	Index case	c.3G>A	p.Met1Ile
					c.-44G>A Met1Ile	NA

Abbreviations: AML, acute myeloid leukemia; CML, chronic myeloid leukemia; MDS, myelodysplastic syndrome; NA, not available; NV, does not have the variant.
