# Supplementary material for: Germline heterozygous DDX41 variants in a subset of familial myelodysplasia and acute myeloid leukemia
Source: Leukemia. 2016 May 20;30(10):2083–6. doi: 10.1038/leu.2016.124 (PMC5008455; doi:10.1038/leu.2016.124)
Supplement: Supplementary Figure S1 [file leu2016124x1.doc]

**Supplemental figure S1**

**Figure S1.** Telomere lengths are slightly shorter in affected individuals (p<0.05). Telomere lengths were measured by monochrome multiplex quantitative PCR.
